# Comparison of Bile Acids and Acetaminophen Protein Adducts in Children and Adolescents with Acetaminophen Toxicity

**DOI:** 10.1371/journal.pone.0131010

**Published:** 2015-07-24

**Authors:** Laura James, Ke Yan, Lisa Pence, Pippa Simpson, Sudeepa Bhattacharyya, Pritmohinder Gill, Lynda Letzig, Gregory Kearns, Richard Beger

**Affiliations:** 1 Department of Pediatrics, University of Arkansas for Medical Sciences, Little Rock, AR 72202, United States of America; 2 Arkansas Children’s Hospital Research Institute, Little Rock, AR 72202, United States of America; 3 Medical College of Wisconsin, Milwaukee, WI 53226, United States of America; 4 Division of Systems Biology, National Center for Toxicological Research, Jefferson, AR 72079, United States of America; 5 Division of Pediatric Pharmacology, Medical Toxicology and Therapeutic Innovation, The Children’s Mercy Hospital, Kansas City, MO 64108, United States of America; UFMG, BRAZIL

## Abstract

Metabolomics approaches have enabled the study of new mechanisms of liver injury in experimental models of drug toxicity. Disruption of bile acid homeostasis is a known mechanism of drug induced liver injury. The relationship of individual bile acids to indicators of oxidative drug metabolism (acetaminophen protein adducts) and liver injury was examined in children with acetaminophen overdose, hospitalized children with low dose exposure to acetaminophen, and children with no recent exposure to acetaminophen. Nine bile acids were quantified through targeted metabolomic analysis in the serum samples of the three groups. Bile acids were compared to serum levels of acetaminophen protein adducts and alanine aminotransferase. Glycodeoxycholic acid, taurodeoxycholic acid, and glycochenodeoxycholic acid were significantly increased in children with acetaminophen overdose compared to healthy controls. Among patients with acetaminophen overdose, bile acids were higher in subjects with acetaminophen protein adduct values > 1.0 nmol/mL and modest correlations were noted for three bile acids and acetaminophen protein adducts as follows: taurodeoxycholic acid (R=0.604; p<0.001), glycodeoxycholic acid (R=0.581; p<0.001), and glycochenodeoxycholic acid (R=0.571; p<0.001). Variability in bile acids was greater among hospitalized children receiving low doses of acetaminophen than in healthy children with no recent acetaminophen exposure. Compared to bile acids, acetaminophen protein adducts more accurately discriminated among children with acetaminophen overdose, children with low dose exposure to acetaminophen, and healthy control subjects. In children with acetaminophen overdose, elevations of conjugated bile acids were associated with specific indicators of acetaminophen metabolism and non-specific indicators of liver injury.

## Introduction

Acetaminophen (APAP) overdose is a major cause of acute liver failure and acute liver injury in the western world [1]. Hepatic metabolism of APAP is known to be a critical factor in the development of hepatotoxicity. Following low dose exposure, APAP is primarily metabolized through conjugation reactions, and oxidation reactions play a relatively minor role. However, following exposure to large doses of APAP, conjugation pathways are saturated and a relatively greater proportion of the drug undergoes oxidative metabolism. Oxidation, which occurs through the cytochrome P450 enzymes located in the centrilobular regions of the liver, generates the reactive metabolite *N*-acetyl-*p*-benzoquinone imine (NAPQI), which, along with the depletion of hepatic glutathione, is recognized to be a critical initiating step in the development of hepatotoxicity [2]. NAPQI binds to cysteine groups on proteins to form APAP protein adducts, which are released from the centrilobular hepatocytes, in addition to alanine aminotransferase (ALT) and aspartate aminotransferase (AST), and enter the peripheral circulation during hepatocyte lysis [3]. Previous experimental and clinical studies suggest that serum levels of APAP protein adducts can serve as biomarkers of APAP-related hepatotoxicity, reflecting the oxidative metabolism of the drug [4–6]. Other mechanisms are known to contribute to the toxicity and include oxygen and nitrogen stress [7], mitochondrial permeability transition [8,9], and intracellular signaling mechanisms involving c-Jun N-terminal protein kinase activation [10].

Over the last decade, “omics”-based approaches have been increasingly used to investigate mechanisms of drug induced liver injury, including APAP toxicity. Metabolomics-based approaches have been used to examine the relationship of intermediates of fatty acid oxidation, energy production, and redox balance to the overall toxicity response [11–14]. For example, Clayton *et al*. showed that variability in the diet could influence conjugation reactions and modulate the overall toxicity response in the rat model of APAP toxicity [11]. In addition, metabolomic approaches have further demonstrated the role of mitochondrial dysfunction in APAP toxicity by showing the elevation of circulating long chain acylcarnitines, substrates of β-oxidation, in experimental and clinical studies [13,15–17].

Alteration of bile acid transport is a known mechanism of drug induced liver injury [18,19]. Elevations of bile acids have been reported for a number of hepatotoxins regardless of the specific pattern of liver injury (necrosis, steatosis, cholestatic, and idiosyncratic). In experimental models of drug induced liver injury, the most significant alterations in bile acid homeostasis were shown for toxins that cause either necrosis or cholestasis [20]. In addition, in mice treated with toxic doses of APAP, it was shown that dietary modulations that alter the existing bile acid pool changed the sensitivity of mice to APAP toxicity [21].

In the following study, targeted bile acid analysis was performed in serum samples from children and adolescents with APAP overdose, healthy children with no recent APAP exposure and hospitalized children receiving APAP per standard of care. Bile acids were compared to APAP protein adducts, an indicator of the oxidative metabolism of APAP [6,22] and ALT, the most widely used clinical indicator of liver injury.

## Patients and Methods

The study was a multicenter study of APAP toxicity in children ages 2–18 years and was approved by the institutional review boards of all participating institutions, in accordance with the guidelines of the 1975 Declaration of Helsinki. Written, parental consent was obtained for all participating subjects and written assent was obtained in subjects over 7 years of age. Informed consent (and assent) documents were approved by the UAMS Institutional Review Board as well as the institutional review boards of all participating sites: Akron Children's Hospital Institutional Review Board, Baylor College of Medicine Institutional Review Board, Children's Mercy Hospital Institutional Review Board, Children's National Medical Center Institutional Review Board, Cook Children's Hospital Institutional Review Board, ProMedica Health Systems Institutional Review Board, and The University of Louisville Institutional Review Board. Following informed consent and assent when age appropriate, blood samples were collected from study subjects classified into three subject groups defined as Group A (Therapeutic APAP dose, consisting of hospitalized children with various medical conditions receiving APAP per standard of care); Group B (Controls, healthy children with no use of APAP in the preceding 14 days); and Group C (APAP Overdose, consisting of children requiring hospitalization for treatment of APAP overdose, based on an assessment of history of ingestion and quantitation of APAP in peripheral blood [23]). For subjects in Group A, timed blood samples were collected prior to receipt of the first APAP dose “on study” and thereafter at 8 and 24 h after the first dose of APAP, followed by convenience sampling throughout the period of hospitalization. A single blood sample was collected in Group B subjects and admission and daily morning blood samples were collected in subjects in Group C. Blood samples were centrifuged within 30 minutes of collection and the serum was stored at -80°C.

### Clinical data collection

Clinical and demographic data were collected prospectively using a study-specific electronic data base that included subject age, gender, weight, height, body mass index, past medical history, and relevant history concerning recent APAP dosing and concomitant medications. Data on dose (mg/kg), frequency, route of administration (oral, rectal, intravenous) were collected for APAP (Group A) and for N-acetylcysteine (NAC), the clinical antidote for APAP overdose (Group C). Clinical laboratory data recorded in the data base included determinations of APAP and ALT performed by participating hospital clinical laboratories. APAP and NAC dosing, route, and frequency were at the discretion of the treating physician.

### APAP protein adduct assay

APAP protein adducts in serum were analyzed using a previously published and validated assay [6,22,24]. Serum was gel filtered using 96 well desalting plates (Pierce, Rockford, IL) and hydrolyzed with protease (Sigma, St Louis, MO) to release APAP-cysteine from APAP protein adducts [6,22]. After precipitation and extraction, samples were injected onto a high performance with liquid chromatography system (ESA Corp, Chelmsford, MA) and resolved on a 150 mm C_18_ column (Symmetry, Waters, Milford, MA). APAP cysteine was detected using a coulometric electrochemical detector and quantified relative to a standard curve of authentic APAP cysteine using Coularray Software (Thermo Scientific, Chelmsford, MA). Final APAP cysteine adduct values were reported as nmol APAP protein adduct/ml serum.

### Metabolomic analysis of bile acids

Targeted metabolomic analysis of nine bile acids was performed using ultra performance liquid chromatography with triple quadrupole mass spectrometry as previously described [25]. Bile acids measured included Cholic acid (CA); Chenodeoxycholic acid (CDCA); Deoxycholic acid (DCA); Taurocholic acid (TCA); Taurodeoxycholic acid (TDCA); Tauchenodeoxycholic acid (TCDCA); Glycocholic acid (GCA); Glycodeoxycholic acid (GDCA); Glycochenodeoxycholic acid (GCDCA). Serum samples (50 μl) were thawed and spiked with isotope labeled individual bile acid standards. The samples were then de-proteinized, dried, reconstituted in methanol:water (50:50, v/v) and subjected to UPLC separation using an Acquity UPLC system (Waters, Milford, MA) equipped with an Acquity UPLC BEH C18 column (2.1 × 100 mm, 1.7 μm particle size). A Waters Xevo Triple quadrupole mass spectrometer operated in ESI-negative mode was used for absolute quantitation of bile acids in serum using stable isotope standards. Multiple Reaction Monitoring transitions for bile acids were optimized by direct infusion of standards.

### Statistical analysis

Non-parametric statistical tests (Kruskal-Wallis) were used to compare clinical and laboratory data among the three groups of subjects (p<0.05) and the Mann-Whitney test was used for pairwise comparisons between two groups (p<0.05). Pearson’s correlation coefficients were calculated for comparison of peak measurements of bile acids to peak APAP protein adducts. Receiver operator curve (ROC) analysis was conducted to identify “cut points” of bile acid measurements that best discriminated subject samples with peak APAP protein adduct values of ≥ 1.0 vs. peak adduct < 1.0 nmol/mL, a previously determined “toxicity threshold” for APAP protein adducts in patients with APAP liver injury [6,24].

## Results

### Clinical and demographic data

A total of 88 children (Group A, 15; Group B, 19; Group C, 64) completed the study. Demographic and clinical data characteristics by group are summarized in Table 1. Children in Groups A and C were older than those of Group B and had higher body weights. More females (68.37%) than males participated in the study as a whole, but there were more males (73.33%) than females in Group A. No significant differences in race were detected among the groups (data not shown), but there were more Hispanics in Group B (5 of 15; p = 0.033), compared to Group A (0 of 19) and Group C (5 of 63).

**Table 1:** Demographic characteristics of study subjects by groups.

Group (N)	APAP Therapeutic Exposure Group A (15)	Healthy Control Group B (19)	APAP Overdose Group C (64)	Global p-value*	A vs. B**	A vs. C**	B vs. C**
Age (years)*	14.08 (2.00, 18.08)	9.33 (2.67, 16.17)	15.54 (1.50, 18.25)	<0.001	0.042	0.189	<0.001
Weight (kg)*	60.90 (11.60, 98.70)	40.80 (11.40, 99.80)	63.55 (10.00, 117.20)	0.001	0.218	0.187	<0.001
Gender (% Male)	73.33	31.58	21.88	<0.001	0.016	<0.001	0.385

Data are presented as median and range.

*p value for three way comparison and **p value for pairwise comparisons.

The median (range) daily dose of APAP for subjects in Group A was determined for the initial 24 and 48 h of the study. The daily dose of APAP was higher (p = 0.014) in the initial 24 h (31.0 [6.2–100.6] mg/kg APAP) compared to the initial 48 h (20.5 [3.1–57.5] mg/kg APAP), indicating a decline in the median daily dose between study day 1 and day 2. Daily doses were within the recommended dose range for APAP use in children [26].

APAP overdose patients may be relatively asymptomatic during the initial 24 hours following APAP overdose, as the development of overt toxicity may not be apparent until 24 to 48 hours after the time of ingestion. Thus, patients are evaluated for the potential risk of hepatotoxicity through the use of a nomogram (ie, Rumack nomogram [27]) based on quantified levels of APAP plotted as a function of the time elapsed since the occurrence of the APAP ingestion. Among the 64 Group C patients, 40 were judged to be “at risk” for liver injury on the basis of the Rumack nomogram [27]. Additional clinical factors which prompted hospitalization and NAC treatment were the elevation of ALT values at the time of presentation to the hospital (n = 5 subjects), and the co-ingestion of antihistamines (n = 2 subjects), which may alter gastric motility and thus limit utility of the nomogram [28]. The time of the ingestion could not be determined for one subject. Thus, overall, 73% of Group C patients were viewed to be “at risk” for the development of liver injury. There were no deaths or liver transplants in the study cohort.

### Group C Treatment Data

All children in Group C received treatment with NAC. Since the efficacy of NAC in preventing hepatic injury is a function of the time it is administered relative to the time of the drug overdose [29], the data analysis was stratified by time to the first dose of NAC relative to the reported time of the APAP ingestion. Time data was known for 60 of 64 subjects in Group C. Distribution of time to first dose of NAC was <10 h for 34 (56.7%) subjects, 10–24 h for 12 (20%) subjects, and ≥24 h for 14 (23.3%) subjects, and was consistent with published studies in APAP toxicity in children [30]. Eleven of the 64 (17.2%) subjects in Group C had ALT values > 1000 IU/L.

### Toxicity and metabolism data

Table 2 provides summary data of descriptive statistics for serum ALT (IU/L) and serum APAP protein adducts (nmol/mL). Since multiple measures were obtained for the study subjects in Groups A and C, the data were analyzed by peak measured value for ALT and APAP protein adducts. Peak ALT differed among the three groups (p = 0.003) and was higher in Groups A (p = 0.003) and C (p = 0.001) than in Group B. However, peak ALT for Group C was not higher than Group A, likely as a function of the large subset of patients within Group C that received early treatment with NAC and thus did not develop toxicity. In contrast, APAP protein adducts differed among the three groups and were statistically different between each group.

**Table 2:** Summary data for peak alanine aminotransferase values (ALT), acetaminophen (APAP) protein adducts, and bile acids by group.

	Therapeutic Exposure Group A	Healthy Control Group B	Overdose Group C	p-value*	A vs. B**	A vs. C**	B vs. C**
ALT (IU/L)	40.00 (7.00, 191.00)	16.00 (10.00, 37.00)	29.00 (8.00, 9909.00)	**0.003**	**0.003**	0.965	**0.001**
Adduct (nmol/mL)	0.16 (0.01, 2.11)	0.006 (0.00, 0.01)	0.30 (0.03, 7.92)	**<0.001**	**<0.001**	**0.005**	**<0.001**
TCA (uM)	0.23 (0.02, 5.22)	0.35 (0.004, 1.46)	0.06 (0.005, 8.70)	**<0.001**	0.238	***0*.*027***	**<0.001**
GCA (uM)	0.45 (0.14, 9.55)	0.15 (0.03, 1.19)	0.21 (0.01, 15.59)	***0*.*018***	**0.007**	***0*.*021***	0.248
GDCA (uM)	1.18 (0.36, 4.60)	0.19 (0.008, 1.85)	1.04 (0.08, 16.52)	**<0.001**	**<0.001**	0.866	**<0.001**
TDCA (uM)	0.31 (0.05, 4.07)	0.05 (0.005, 0.93)	0.24 (0.02, 12.04)	**<0.001**	**<0.001**	0.263	**<0.001**
CA (uM)	1.34 (0.18, 18.26)	6.41 (0.34, 49.64)	0.61 (0.03, 10.42)	**<0.001**	**<0.001**	0.100	**<0.001**
CDCA (uM)	0.07 (0.009, 1.03)	0.20 (0.00, 0.54)	0.17 (0.03, 3.46)	0.066	0.252	***0*.*017***	0.540
DCA (uM)	0.19 (0.06, 2.47)	0.09 (0.005, 2.52)	0.29 (0.02, 3.90)	0.058	0.056	0.750	***0*.*024***
GCDCA (uM)	0.10 (0.00, 3.21)	0.04 (0.003, 0.26)	0.19 (0.02, 3.69)	**<0.001**	0.238	0.132	**<0.001**
TCDCA (uM)	0.16 (0.00, 2.29)	0.12 (0.004, 0.74)	0.15 (0.008, 4.02)	0.596	0.425	0.528	0.451

Data presented as median (range).

*p value for three way comparison.

**p value for pairwise comparison.

Bold p values indicate significance p<0.01; bold, italicized p values represent p = 0.01–0.05.

### Bile acid metabolites

Summary data, presented as peak values, for bile acids by subject group are presented in Table 2. Significant differences were detected among the three groups for serum levels of 6 of the 9 bile acids (excluding TCDCA, CDCA, and DCA, **three way comparison). As shown in Table 2, pair-wise comparisons between groups showed that the greatest differences in bile acids were observed for subjects in Group C (overdose) compared to Group B (healthy controls). TDCA, GDCA, and GCDCA were elevated in Group C compared to values in healthy controls. In contrast, TCA and CA were decreased in Group C compared to Group B (healthy controls). However, comparison of bile acids in Group C (overdose) to Group A (therapeutic exposure) revealed fewer differences among the bile acids. Only CDCA was higher in Group C than in Group A subjects (p<0.05), while TCA and GCA were lower in Group C than in Group A. Comparison between Groups A and B showed greater variability among bile acids in Group A than Group B, possibly as a function of the heterogeneous nature of disease among the subjects in Group A.

### Comparison of bile acids to toxicity parameters

**Fig 1:**
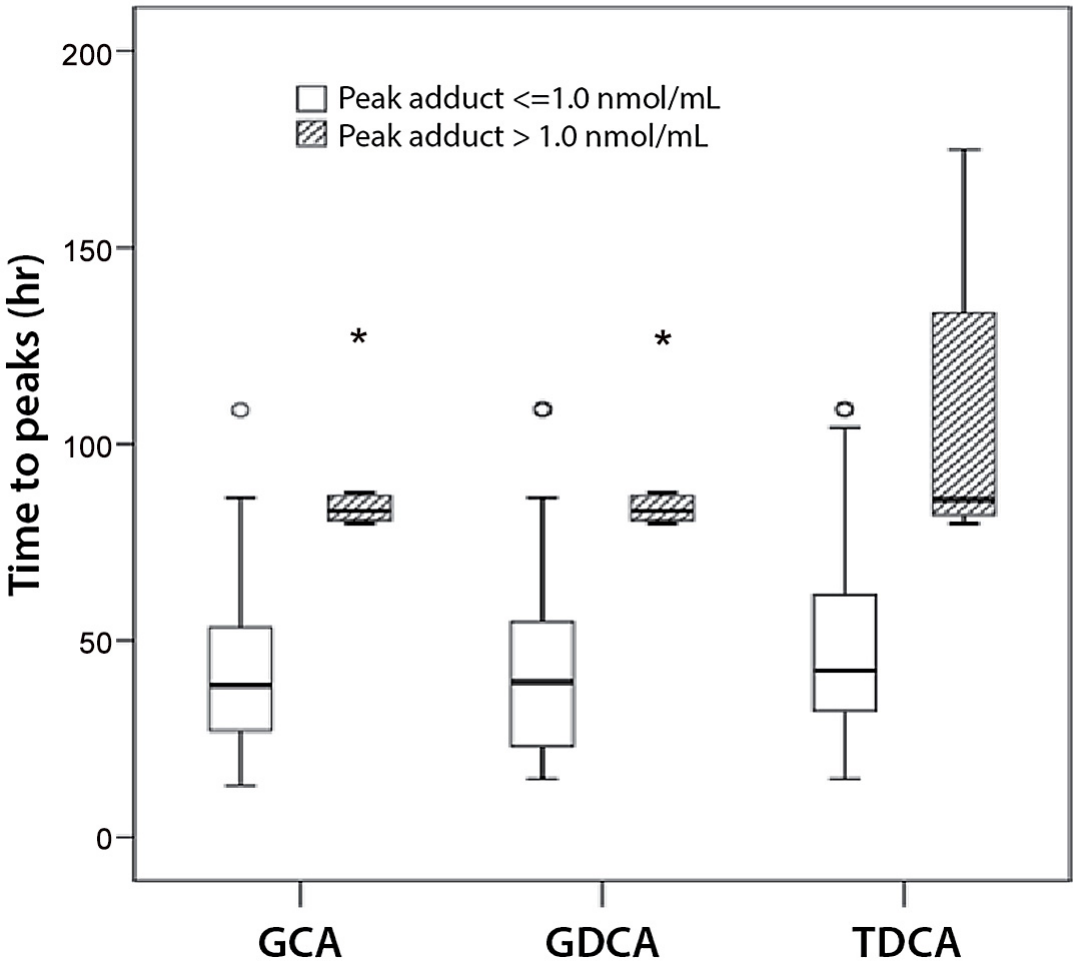
Comparison of time to reach peak bile acid, presented as a function of peak APAP protein adduct < or ≥ 1.0 nmol/mL APAP protein adduct. *denotes > 2.0 times the 25–75^th^ percentile; ^O^denotes > 1.5 times the 25–75^th^ percentile. GCA, Glycocholic acid; GDCA, Glycodeoxycholic acid; TDCA, Taurodeoxycholic acid.

Due to the limited subject sampling scheme of this pediatric study, relative comparison of temporal changes in bile acids, APAP protein adducts and ALT in the early hours after the APAP overdose was not possible. As an alternative, the time to reach peak measurement for individual bile acids was examined and compared as a function of APAP protein adduct concentrations. APAP protein adduct values ≥ 1.0 nmol/mL have been shown to have high sensitivity and specificity (97 and 95%, respectively) in patients with APAP liver injury (defined as an ALT value of > 1000 IU/L) [6,24]. As shown in Fig 1, time to reach peak measure for each bile acid was shorter in subjects with APAP protein adducts < 1.0 nmol/mL, compared to subjects with APAP protein adducts ≥ 1.0 nmol/mL. Correlation analysis was also performed for peak APAP protein adducts versus peak bile acid levels. The highest correlations were noted for TDCA (R = 0.604; p<0.001), GDCA (R = 0.581; p<0.001), and GCDCA (R = 0.571; p<0.001).

**Fig 2:**
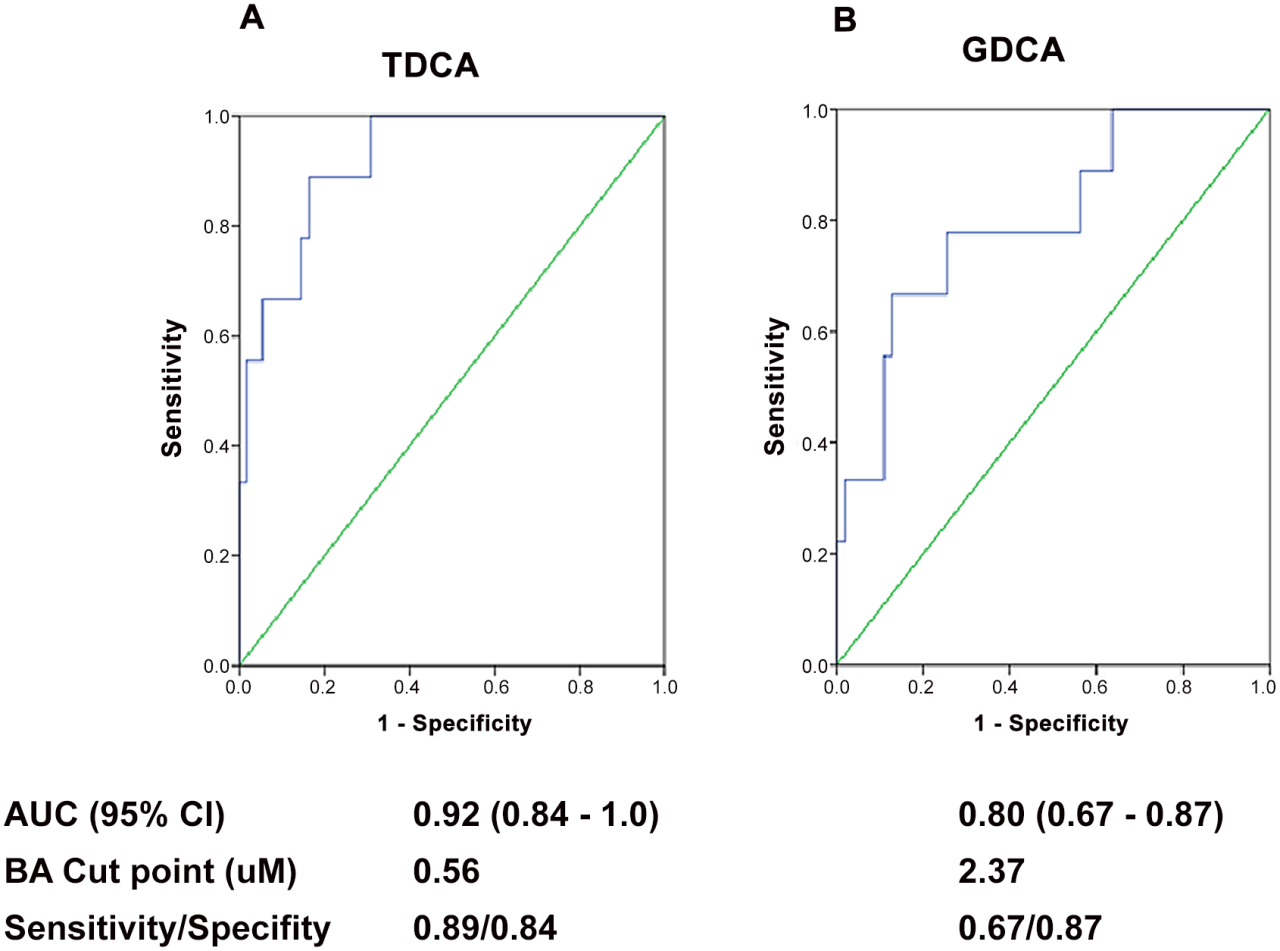
Receiver operator curve (ROC) analysis of bile acids (BA) that best distinguished subjects with acetaminophen protein adducts < 1 versus ≥ 1.0 nmol/mL. TDCA, Taurodeoxycholic acid; GCDA, Glycodeoxycholic acid.

In subsequent analysis, ROC analysis was conducted to identify bile acid cut points that maximized the sum of sensitivity and specificity for discriminating patients with peak adducts < 1.0 nmol/mL or ≥ 1.0 nmol/mL. TDCA values > 0.56 uM had the highest area under the curve value for discriminating subjects at the adduct toxicity cut point of 1.0 nmol/mL (Fig 2), followed by GDCA.

In previous work, APAP protein adducts and ALT were shown to be higher in patients with greater delays in receiving treatment with NAC [31]. Thus, the relationship of bile acids to initiation of treatment with NAC was examined and compared to that of APAP protein adducts and ALT. Table 3 provides correlations between individual parameters in relationship to time to NAC treatment. None of the bile acids were highly correlated with time to NAC treatment; ALT and APAP protein adducts were modestly correlated with time to NAC treatment.

**Table 3:** Correlation analysis of peak biomarker versus time to treatment with N-acetylcysteine (NAC)*.

Parameter vs. Time to NAC*	R value	P Value
ALT	0.569	p<0.001
APAP Protein Adduct	0.489	p<0.001
GDCA	0.421	p<0.001
TDCA	0.397	p = 0.002
GCA	0.372	p = 0.004
GCDCA	0.332	p = 0.010
DCA	0.319	p = 0.013
TCDCA	0.287	p = 0.026
TCA	0.202	p = 0.121
CA	0.201	p = 0.123
CDCA	0.181	p = 0.166

*Log transformation of peak measurement of parameter.

Thus, overall, the data suggest that several of the conjugated bile acids associate with toxicity severity in APAP overdose, as defined by both ALT values and APAP protein adduct concentrations.

## Discussion

Bile acids are formed during the biotransformation of cholesterol through two metabolic pathways known as the “classical” or neutral pathway and the “alternative” or acidic pathway. The classical pathway produces primary bile acids and involves microsomal cholesterol 7α-hydroxylase (CYP7A1), while the alternative pathway involves biotransformation of cholesterol through mitochondrial sterol 27-hydroxylase (CYP27A1) followed by oxysterol 7α-hydroxylase (CYP7B1) [32]. More than 90% of bile acids in humans are derived from the classical pathway, represented by cholic acid (CA) and chenodeoxycholic acid (CDCA), which are conjugated by either taurine or glycine for secretion into bile. Deoxycholic acid is a secondary bile acid that is formed through dehydroxylation by intestinal bacteria and is reabsorbed through enterohepatic recirculation to maintain a constant pool of bile acids [33].

The increased availability of metabolomics technology has enabled investigations of the relationship between small molecules of endogenous metabolism and the development of liver injury in experimental models of APAP toxicity [11,13,25,34]. A 1975 publication found that a composite measure of bile acids was more sensitive than serum AST for detecting APAP liver injury verified by liver biopsy [35]. Using a multicenter, prospective sample collection design, serum bile acids were compared among hospitalized children and adolescents with APAP overdose, low dose APAP exposure, and healthy children with no recent APAP exposure. Multiple differences in individual bile acids were observed among the three groups (Table 2). Elevations of conjugated bile acids (e.g., GDCA, TDCA and GCDCA) were observed in the APAP overdose group compared to the healthy control group. However, differences in the conjugated bile acids were less pronounced between the two APAP exposure groups (Groups A and C), possibly as a function of the disease heterogeneity of the subjects in Group A, or the relatively low morbidity of the pediatric cohort of the study [32]. Conjugated bile acid elevation was associated with the severity of toxicity (Fig 1), which we defined on the basis of APAP protein adduct levels based on our previous data [6,24]. Comparison of bile acids (Fig 2) in patients with an APAP protein adduct level > 1.0 nmol/mL, a previously identified “cut-point” value with high sensitivity and specificity for APAP toxicity in patients with an ALT values above 1000 IU/L, showed modest, but statistically significant correlations between peak measurements of adducts and peak measurements of TDCA (R = 0.604; p<0.001), GDCA (R = 0.581; p<0.001), and GCDCA (R = 0.571; p<0.001). Similar to our study, a recent report examined specific bile acids in adults (median [range] of age, 34 [18–61 years]) with APAP-related acute liver failure and found that GCDCA, TCDCA, GCA, TCA, GDCA, and TDCA were elevated in patients above the values found in controls and that GDCA was significantly higher in APAP non-survivors, compared to survivors [36]. Of note, the authors found that GDCA values were higher in the APAP liver failure group than in patients with cholestatic liver injury. Our study differed from this earlier study in that our study population was younger and had lower morbidity and no mortality and thus comparison of individual bile acids to survival was not possible.

Examination of the *primary* bile acids showed that CA was lower in the APAP overdose group (compared to Group B), while CDCA was only reduced in Group A when compared to Group B. The lower levels of CA in the APAP overdose subjects could be consistent with a protective mechanism of the liver and a shift from the classical to the alternative pathway of metabolism, as has been shown for non-alcoholic fatty liver disease [32].

The mechanistic significance of elevated bile acids in children with APAP overdose and liver injury is unclear. Previous studies in rodent models of APAP toxicity [25,37] found elevations of taurocholic and deoxycholic acid in blood samples of rats treated with APAP and noted correlations among bile acids and serum ALT and among bile acids and necrosis scores [25]. In addition, genes regulating bile acid synthesis (*Cyp7A1* and *Cyp8B1*) and cholesterol transport (*Abcd1*) were down-regulated, while genes that increased bile acid transport (*Mrp2*, *Mrp3*, *Mrp4*) were up-regulated [37]. Thus, data in the experimental model of APAP toxicity (ie., bile acid perturbations and changes in gene regulation) indicated the occurrence of transient intrahepatic cholestasis [25]. During bile acid metabolism, bile acids are conjugated by taurine or glycine at the carboxyl position to form metabolites that are more hydrophilic and thus less toxic to hepatocytes [33]. Treatment with taurine (2-aminoethanesulfonic acid), a conditionally essential organic acid formed as an end-product of methionine and cysteine metabolism [38], was protective in a number of organ injury models, including experimental studies of APAP toxicity [39]. Taurine also has anti-oxidant effects *in vitro* [40]. The taurine conjugates of bile salts (e.g., ursodeoxycholic acid) are hepatoprotective and stimulate bile acid excretion by evoking signaling mechanisms involving intracellular calcium [41]. Thus, the pattern of increased levels of conjugated bile acids in subjects with APAP overdose could be consistent with a regulatory, protective response of the liver. In a metabolomic profiling study conducted in rodents, the upregulation of primary bile acids and their conjugates was observed for toxins that had primarily either a necrotic pattern of liver injury (APAP, bendazac, methapyrilene, and ticlopidine) or a cholestatic liver injury pattern (eg., DL-ethionine) [20]. Bile acids were not increased in response to nephrotoxins or muscular toxins. Thus, it was postulated that bile acids may represent very sensitive markers of drug induced liver injury and be tested as potential candidate biomarkers for future application in liver screening panels.

APAP protein adducts reflect the contribution of oxidative metabolism to APAP toxicity [42] and are specific to APAP exposure [5]. In addition, the sensitivity of the analytical assay for quantitation of APAP protein adducts is such that very low levels of adducts can be quantified in subjects receiving APAP in the clinical setting [43]. As shown in Table 2, differences in adduct levels between “therapeutic” exposure (Group A) and “toxic” (Group C) were apparent in this study. The elevation of bile acids is not specific to APAP toxicity and it is likely that bile acids are elevated in other forms of drug-induced liver injury in man [22], as well as other disease conditions, including NAFLD and cholestatic liver disease. Further study of bile acids as potential predictors of survival, as suggested by others [36], appears warranted. Our data demonstrate that understanding bile acid profiles in the context of other medical conditions and exposure to “low dose” APAP is also an important component of biomarker examination (Table 3). In previous work, we found observed a relationship of peak adduct levels to treatment delay with NAC. [31] The relationship of biomarker profiles to the initiation of treatment with NAC is also an important consideration in studies of biomarkers in APAP toxicity (Table 3).

It has also possible that perturbations of bile acid profiles may be useful in the future to examine the homeostatic integrity of the liver in the recovery and regeneration stages of liver injury. This concept has been tested in a previous clinical study in which bile acid profiles in biliary fluid were examined pre- and post- liver transplant as a functional indicator of metabolic capacity [44]. The limited sampling nature of the present study did not allow full comparison of the predictive toxicity potential of APAP protein adducts versus bile acids. An important question to address in future studies would be whether or not elevation of bile acids proceeded or followed APAP protein adduct elevations and the relative temporal relationships of these biomarkers to ALT elevations in patients with APAP overdose and resulting toxicity.

Several additional limitations of the present study merit consideration. The three subject groups differed somewhat by age and by distribution of gender (Table 2). In addition, the doses of APAP administered in Group A were not controlled by the study and were administered on an as needed basis due to the age group of the study. Group A subjects were heterogeneous with respect to the medical conditions requiring hospitalization which may have contributed to the variability of bile acid profiles in this group. In addition, bile acids may be influenced by diet and other environmental effects. The subjects in Group C represent a previously healthy cohort of patients with minimal pre-existing morbidity and no mortality. A particular advantage of the pediatric population is the relative lack of pre-existing liver disease as would be anticipated to be more common in older populations. In addition, co-ingestions with other drugs, such as opioids, are relatively rare in pediatric APAP toxicity [30], as opposed to adults with APAP overdoses resulting in severe liver injury and liver failure [1].

The data provided herein have application to other studies of drug induced liver injury and suggests a potential role for bile acid profiles as sensitive determinants of liver injury in drug development. Additional research to further understand the mechanistic significance of bile acid elevations in APAP exposure and toxicity is of interest, especially in light of recent studies that have illustrated the role of bile acids as signaling molecules that are important in lipid and glucose metabolism. Finally, this study illustrates how metabolomic approaches can be used as a tool for biomarker discovery and hypothesis generation in the clinical setting of drug toxicity and liver injury.
